# Comparison of family centered care with family integrated care and mobile technology (mFICare) on preterm infant and family outcomes: a multi-site quasi-experimental clinical trial protocol

**DOI:** 10.1186/s12887-019-1838-3

**Published:** 2019-12-02

**Authors:** Linda S. Franck, Rebecca M. Kriz, Robin Bisgaard, Diana M. Cormier, Priscilla Joe, Pamela S. Miller, Jae H. Kim, Carol Lin, Yao Sun

**Affiliations:** 1 University of California San Francisco, School of Nursing, 2 Koret Way, Box 0606, San Francisco, CA 94143 USA; 2 UCSF Benioff Children’s Hospital, San Francisco, 1975 4th St, San Francisco, CA 94158 USA; 3 Community Regional Medical Center, 2823 Fresno St, Fresno, CA 93721 USA; 4 Division of Neonatology, UCSF Benioff Children’s Hospital, Oakland, 747 52nd Street, Oakland, CA 94618 USA; 5 UCLA Health, 924 Westwood Boulevard, Suite 720, Los Angeles, CA 90024 USA; 6 Division of Neonatology, Perinatal Institute, Cincinnati Children’s Hospital Medical Center, 3333 Burnet Avenue, MLC 7009, Cincinnati, OH 45229 USA; 7 Kaiser Permanente Santa Clara, 3rd Floor Dept. 340.710 Lawrence Expy, Santa Clara, CA 95051 USA

**Keywords:** Family integrated care, Neonatal intensive care unit, Mobile application, Family centered care, Parents, Infants

## Abstract

**Background:**

Family Centered Care (FCC) has been widely adopted as the framework for caring for infants in the Neonatal Intensive Care Unit (NICU) but it is not uniformly defined or practiced, making it difficult to determine impact. Previous studies have shown that implementing the Family Integrated Care (FICare) intervention program for preterm infants in the NICU setting leads to significant improvements in infant and family outcomes. Further research is warranted to determine feasibility, acceptability and differential impact of FICare in the US context. The addition of a mobile application (app) may be effective in providing supplemental support for parent participation in the FICare program and provide detailed data on program component uptake and outcomes.

**Methods:**

This exploratory multi-site quasi-experimental study will compare usual FCC with mobile enhanced FICare (mFICare) on growth and clinical outcomes of preterm infants born at or before 33 weeks gestational age, as well as the stress, competence and self-efficacy of their parents. The feasibility and acceptability of using mobile technology to gather data about parent involvement in the care of preterm infants receiving FCC or mFICare as well as of the mFICare intervention will be evaluated (Aim 1). The effect sizes for infant growth (primary outcome) and for secondary infant and parent outcomes at NICU discharge and three months after discharge will be estimated (Aim 2).

**Discussion:**

This study will provide new data about the implementation of FICare in the US context within various hospital settings and identify important barriers, facilitators and key processes that may contribute to the effectiveness of FICare. It will also offer insights to clinicians on the feasibility of a new mobile application to support parent-focused research and promote integration of parents into the NICU care team in US hospital settings.

**Trial registration:**

ClinicalTrials.gov, ID NCT03418870. Retrospectively registered on December 18, 2017.

## Background

Preterm birth is a leading cause of long-term disabilities and costs the US economy well over $26 billion annually [1]. Poor growth during Neonatal Intensive Care Unit (NICU) hospitalization significantly increases a preterm infant’s risk of mortality and serious long-term morbidity [2, 3]. There is strong evidence that preterm infants who receive human milk have better growth and neurodevelopmental outcomes, reduced risk of major morbidities such as necrotizing enterocolitis (NEC), intraventricular hemorrhage (IVH), retinopathy of prematurity (ROP) and bronchopulmonary dysplasia (BPD), lower rates of nosocomial infection, and lower hospital costs [4–7]. Active parent involvement in preterm infant caregiving leads to higher breast feeding rates, earlier discharge and improved long-term neurodevelopment [8–12]. Secure parent-infant attachment and sensitivity to infant cues are essential to long-term quality of life for these infants and are significantly improved by NICU interventions that promote parenting self-efficacy and physical and emotional closeness between parents and infants [13–16]. Conversely, NICU-related parent stress and depression adversely affect preterm children’s long-term social, behavioral, and functional development [17, 18].

### Family-centered care (FCC)

FCC is a philosophy and healthcare delivery framework that recognizes the central role of the family in an individual’s health and well-being. Institutions and professionals that ascribe to FCC principles are expected to meaningfully engage patients and families in healthcare that is culturally and individually tailored and builds self-care skills, knowledge, confidence and shared decision-making [19–22]. FCC in NICUs is inconsistently conceptualized and practiced, resulting in confusion over the degree to which the care delivery models fully incorporate families as parterns in care [23]. Thus, the majority of patient care is delivered by NICU professionals, not parents [24–28]. Parent are still treated as “visitors”, do not view themselves as their infant’s primary caregiver, experience high levels of anxiety and stress, and often feel unprepared to care for their infant after discharge [29–35]. Geographic, racial/ethnic, and sociodemographic disparities in FCC have been reported by parents in the US with respect to the healthcare of their children and in attitudes of the multidisciplinary team [36–38].

### Family integrated care (FICare)

Models of NICU care in which parents are the infant’s primary caregivers have shown positive outcomes for infants and parents in both low and high-income settings [23, 39–41] but none have yet demonstrated sustainability or scalability. The Canadian FICare program is a promising new approach [42]. FICare differs from US-practiced FCC in the following ways:

1) Nurses receive formal education on how to teach parents to safely provide NICU care, and thus the focus shifts from the nurse to the parent as primary caregiver; 2) Parents spend a minimum of six hours per day in the NICU and attend group classes with a formal parent-focused curriculum on how to provide primary care of their infant; 3) Parents are explicitly incorporated into daily medical rounds, report on their infant and share in the clinical decisions; and 4) Trained “alumni” parents of former NICU infants provide support to the current NICU parents [42–45].

### Previous studies of FICare

A 25-site cluster randomized trial (cRCT) across Canada (*n* = 18), Australia (*n* = 6) and New Zealand (n = 1) found that in NICUs randomized to FICare (*n* = 13; 827 infants), infants had significantly improved 21-day weight gain (primary outcome) and a greater proportion of them received breast milk at discharge compared with usual care sites (*n* = 12; 873 infants) [46]. There was also lower maternal stress and anxiety in the FICare group. There were no significant between-group differences in the main neonatal morbidities (NEC, ROP, BPD). Analysis is ongoing for other outcomes. These results are compelling; however, FICare implementation required significant day-time involvement on the part of parents. The refusal rate based on time commitment required was approximately 43%. Moreover, more Caucasian mothers and those working outside the home were enrolled in the FICare group, suggesting a selection bias perhaps linked to the time commitment required.

Results from a prospective parallel case-control study of FICare in China [47] showed significantly increased breastfeeding rates, breastfeeding duration, enteral nutrition duration, and weight gain at discharge [48], and higher scores on the mental development index and psychomotor development index at 18 months [49]. Of note, the FICare protocol in this study did not include peer-to-peer support from parents of former preterm infants and required parents to be in the hospital 3 h per day instead of 6 h per day. A FICare-based intervention bundle called Integrated Family Delivered Neonatal Care (IFDC) in the UK was shown to reduce overal length of stay and special care days and to shorten the time to full suck feeding compared with historical controls [50]. The IFDC bundle differed from FICare in that parents received additional support from IDFC coordinators and access to a free mobile app with educational information and a diary. Parents did not receive peer-to-peer support.

Additional studies are being conducted in Level II NICUs in Canada to evaluate length of stay, infant and maternal clinical outcomes, and cost [51] including breastfeeding self-efficacy and breastfeeding rates at discharge [52] with a lower acuity population.

### Knowledge gap

There are many similarities, but also fundamental differences between US and Canadian health and social care systems that might impact the feasibility and outcomes of FICare. For example, US families do not have the same statutory paid parental leave benefits as Canadian parents [53] and have greater out of pocket expenses and healthcare administrative burden [54]. There is also evidence of differences in the composition of the NICU workforce, amount of NICU resources, and degree of parent participation in some types of decision-making between the countries [55, 56]. Further innovation in the FICare program is clearly needed for the program to be feasibly and equitably implemented in US NICUs, particularly to improve accessibility of FICare for parents who live at a distance from the hospital, or have childcare or employment issues, and other significant social stressors. Another important knowledge gap is about the mechanism of action or relative efficacy of the FICare program components. The Canadian-led cRCT [46] was not designed for fine-grain analysis of the individual components of FICare to determine which were most strongly associated with the outcomes of interest overall or for specific subgroups. Further research will help better tailor the intervention to individual parent or infant characteristics, and to evaluate the post-discharge effects.

### Technology enhancement

Mobile technology to engage and support US parents is rapidly advancing and may improve feasibility and accessibility of the FICare program. A systematic review of mobile health interventions for parents in neonatal intensive care units [57] found only eight studies, with the majority being of low or very low quality. The review found no clear impact on neonatal outcomes and no meta-analysis was conducted due to the heterogeneity of the studies. In addition to the IDFC app, we found one federally funded exploratory study of mobile technology to support parents of preterm infants, with that study focused solely on the transition from hospital to home [58]. Use of mobile technology with more interactive features in concert with FICare may enable greater parent participation in FICare when parents cannot be physically present in the NICU.

In partnership with parents and NICU healthcare professionals, we developed the We3health™ app to serve as both a data collection platform for FCC and FICare research and to supplement the delivery of FICare program content for parents, especially for those who are unable to be present in the NICU during daytime hours. The present study will be the first known implementation and research of the Canadian FICare program in the US, with the added innovation of a mobile application, We3health™, to facilitate parental education, clinical communication, parental support, and data collection.

## Methods and design

### Aims

The primary aim is to determine the feasibility and acceptability of mobile application technology to gather data about parent involvement in the care of NICU infants in the usual FCC and in the mFICare parent intervention.

The secondary aim is to compare usual FCC with mFICare on growth and clinical outcomes of preterm infants < 33 weeks gestational age, as well as the stress, competence and self-efficacy of their parents. The analysis will:

1) estimate the effect size for infant growth (primary outcome), defined as change in weight (z-score) at 21 days of age after enrollment, between the usual FCC and mFICare groups; and 2) estimate effect sizes for infant and parent outcomes (secondary outcomes). Infant clinical outcomes at NICU discharge include: weight gain velocity, rates of breastfeeding, nosocomial infection, necrotizing enterocolitis, intraventricular hemorrhage, retinopathy of prematurity, adverse events, and length of stay. Secondary parent outcomes include: perceived stress, parenting competence and parenting self-efficacy. Three-month post-discharge outcomes include: infant growth, breastfeeding, readmission rates and perceived parenting self-efficacy.

### Hypotheses

The primary hypothesis is that the We3health™ mobile application will be feasible and acceptable, capturing high-quality data about the degree of parent involvement and the parent experience in FCC and mFICare conditions and enabling parents to access FICare content to supplement the in-person FICare interventions as well as extend access to FICare interventions for parents who cannot participate in some or all of the in-person FICare program. We secondarily hypothesize that participation in mFICare will be more effective than usual FCC in improving infant and parent outcomes at discharge and post-discharge within and across sites.

### Preliminary work

We gathered extensive input from parents and front-line NICU healthcare professionals to inform the design of this research. We worked closely with parents of premature infants, the multidisciplinary team (nurses, physicians, respiratory therapists, social workers, psychologists, chaplains), and industry consultants in design thinking and mobile technology to adapt the FICare program to the US context. With support from the alumni parent-founded Will’s Way Foundation and the UCSF Center for Digital Health Innovation, we developed the secure HIPAA-compliant We3health™ mobile application to engage parents in key elements of the FICare program and to capture high quality data about parent involvement for all study participants with minimal burden. We have conducted preliminary user testing with NICU parents. All parents found the We3health™ useful and gave positive feedback about the features. We engaged the multidisciplinary team in the NICU on how best to implement each component within their specific NICU context, including establishing nurse-led workgroups to facilitate implementation and address challenges throughout the study.

### Study design

We will use an exploratory prospective sequential cohort quasi-experimental study design to assess the feasibility, acceptability and effect of mFICare in the NICU on infant and parent outcomes. Sequential enrollment will mitigate the between-group contamination or perceived inequities in quality of care that would likely occur with concurrent group enrollment at a single site. This design will resemble most closely the conditions of any single institution in a future cRCT. A parent/infant pair (primary pair) will be eligible for the study. Secondary pairs, for example a twin infant and the other parent from the same family, may be enrolled. Secondary pairs will not be included in the primary analysis but may be included in secondary analyses.

### Setting

The study will be conducted in six NICUs throughout California. The multi-site design enables exploration of implementation barriers and facilitators and potential to scale the interventions across a variety of NICU types, including academic medical centers, freestanding children’s hospitals, and community medical centers and with inborn and transported infants. The sites are also geographically and demographically diverse. A list of the participating sites can be found here [59].

### Participants and eligibility

Parents (guardians) of infants born at 33 weeks gestation or less will be invited to participate in the trial. Participants will be excluded if: (1) the parent is not English literate, is less than 18 years of age, or does not have access to a hand-held computer (smart phone or tablet); or (2) the infant has a life-threatening congenital anomaly, is so critically ill that s/he is unlikely to survive, or is receiving palliative care. Enrollment is limited to English literate parents because the We3health™ version used for this study is only available in English.

### Recruitment

Parent/infant primary pairs will be enrolled sequentially into one of two groups, usual FCC (Phase 1), then mFICare (Phase 2), at one of the six study sites. We aim to recruit 200 pairs for Phase 1 and 175 for Phase 2. The study has been approved by the institutional review boards at each site.

Parents of eligible infants will be given a study information sheet by clinical staff caring for the infants and, if interested in learning more, they will meet with a member of the research team to revew the informed consent information. This information will describe the study, including the different ways parents/guardians may participate, and the risks, benefits, and alternatives of each type of participation, and that they can withdraw from the study at any time without penalty to themselves or their infant’s care. Those who agree will give their written informed consent to participate. Parents may request to defer approach or enrollment to a later date, as long as the infant is expected to remain in the NCU for a minimum of 21 days.

For multiple births, the primary infant will be selected by random assignment. Statistical staff from the university have prepared computer-generated random number schemes for twin and triplet births and placed the assignments in opaque envelopes numbered sequentially. Study site staff are blinded to the randomization schemes. Once a parent has signed the consent form, the study staff will retrieve the appropriate envelope in the sequence and open it to reveal which of the infants will be the primary infant for the study. If there are two parents/guardians who meet eligibility requirements, and both are eligible to participate, the parent who expects to spend the most time in the NICU will be designated as the “primary” pair for the primary analysis. Additional parent-infant pairs from the same family are non-independent and therefore are excluded from the primary analyses. Once a parent has consented to be involved in the trial, they will be assigned a study ID number and contact information will be collected. The parent will be sent an invitation to download and register on We3health™. Parents will receive a $25 gift card at discharge, upon completion of all study questionnaires, and another $25 gift card after completion of the 3-month post-discharge questionnaire.

Nurses and physicians at the sites will be recruited to complete a questionnaires during the FCC and mFICare periods. They will receive an email explaining the project, risks, benefits and alternatives, and inviting them to complete the study questionnaires online. Their completion of the surveys indicates their consent to participate. Follow-up invitations will be sent to non-responders every two weeks until the questionnaires have been returned, or until a total of three reminder messages have been sent. Survey participants will be entered in a drawing to win one of four $25 gift cards during each study phase.

### Intervention

The usual FCC group will be recruited first. Data will be collected for this group without any change in the usual NICU practice. Recruitment for the mFICare group will begin after a break to allow any parents in the NICU who were in the FCC group to complete their study activities (about 2 months).

#### Usual FCC group (Phase 1)

##### Education

Parents will receive an orientation to the unit by a NICU nurse and will have access to the usual written and video materials provided by the NICU. Parents will receive all NICU- required individualized parent teaching and support, delivered at the bedside by nurses, the Discharge Coordinator, other specialists, or in a discharge class.

##### Direct care

Parents will be encouraged by nurses to participate in infant care under nursing supervision for feeding, bathing, dressing, and holding skin-to-skin. Social work and other support: Individualized support from social workers, developmental specialist, lactation consultants, physical therapy, occupational therapy, and other specialists will be offered.

##### Documentation

Parents will not be asked to document any observations of their infant or their own skills acquisition. Nurses and Discharge Coordinators will complete the standard Discharge Teaching Checklist per usual hospital policies. Parents will be asked to use a version of the We3health™ mobile application with limited features to track their time in the NICU, time learning and time spent in infant caregiving activities and to keep of a journal of their NICU experience. These data will not be shared with the clinical team.

#### mFICare group (Phase 2)

##### Clinical team and alumni parent training

After completion of the usual FCC group enrollment, nurses who volunteer to provide care to mFICare infants/parents and the volunteer alumni parents will receive in-person and online training from the study team. The nurse training will follow the Canadian FICare curriculum. Alumni parent training will follow the Canadian FICare curriculum [43]. Physicians, therapists and social workers will receive in-person and online in-service education specific to their roles.

All elements of the FICare model are provided in-person. Additionally, education, daily medical rounds, peer support and documentation are supported by the We3health™ app so that parents may access these components of the intervention at other times than offered in person or when they are away from the NICU.

##### Education

Participants will be oriented to the unit by an mFICare nurse who will introduce them to the program, explain the parent’s role as primary caregiver for their infant, and orient them to the NICU and parent resources, including We3health™. Parents will be provided the Parent Education program, a 3-week rotating curriculum offered a minimum of 3 afternoons per week [43, 46]. The small group sessions will be facilitated by a member of the study team, clinical staff, or alumni parents. Parents will participate in-person or access the content remotely at a time of their choosing via We3health™.

##### Direct care

Parents will be treated as the infant’s primary caregiver, with nurses serving as teachers and coaches. Parents will not be required to be in the NICU at specified hours as in the Canadian FICare program, but when in the NICU, they will be expected to provide as much infant care as they can, with support from mFICare-trained nurses. They will not provide ventilation management, intravenous fluid or intravenous medication administration.

##### Daily medical rounds

Parents will be encouraged and supported to participate in daily medical rounds either in-person or remotely. Parents will report standardized data of their infant’s status to the rounding care team ask questions, and reach consensus with the clinicians on the infant’s daily plan. Nurses will provide role-modeling and coaching to prepare parents for this role. Parents may begin by simply introducing themselves and their baby to the team and gradually increase their level of participation over time.

##### Peer Support

Parents will be offered peer-to-peer support from alumni parents who will contact them remotely, by telephone, text or through We3health™, at least twice weekly and as desired. Each site will build upon its existing peer support program or will be assisted by the study team to establish a new program. The sites will determine their selection criteria for alumni parents.

##### Documentation

Parents will be expected to document time spent with their infant and record infant caregiving (eg skin-to-skin and feeding), observations and skills acquisition using We3health™. They will also use We3health™ to keep a journal of observations of their infant and their own NICU experiences, which they can share with family, friends and the clinical team if they wish via email or social media links. Parents will access the mFICare skills checklist via We3health™ and track their learning and skills acquisition. The infant observation data, parent-team communication and parent skills will be accessible to the clinical team and parents for rounds.

#### Parent resources for both groups

All parents, regardless of group assignment, will be provided with similar resources allowing for them to spend extended periods with their infant, facilitating breast feeding, and providing support for parents that may include reclining chairs, family lounges, kitchens, locked storage, wifi, and others. Resources may vary by site.

### Data collection

#### Clinical data

Infant electronic medical record (EMR) data will be collected by study staff and include: demographics, antenatal/obstetric risks, delivery complications, admission illness severity scores, major clinical treatments (e.g., respiratory support, surgical procedures), adverse events, discharge diagnoses, disposition and length of stay.

#### Parent survey data

Parents will receive four sets of surveys – at enrollment (Baseline), 3 weeks after enrollment, at discharge, and at 3 months post-discharge (Table 1). Questionnaires for parents will be delivered via the We3health™. Baseline surveys will be delivered over 3 days to minimize survey burden. 3-week surveys will be delivered over 4 days. Paper questionnaires will be provided upon request. At 3 months post-discharge, parents will be sent an email with a link to secure online questionnaires or will be mailed questionnaires, depending on preference. Research assistants will be available to parents in both groups to support and encourage completion of the study surveys and We3health™ data.

**Table 1:** Parent measures

Scale	Baseline	21 days after enrollment	Discharge	Post-discharge
Demographics^1^	X			
State & Trait Anxiety [60]	X	X	X	X
Parent Stressor Scale: NICU [32, 61]	X	X		
Family Centered Care [62]	X		X	
Perceived Parenting Self-Efficacy Tool (PMSE) [63]		X	X	X
Nurse-Parent Support Tool [64]		X		
Readiness for Hospital Discharge Survey [65]			X	
mFICare Parent Survey (mFICare group only)			X	
Growth, Feeding current health^1^				X
Major life events^1^				X
What being the parent of a baby is like (WPL-R) [66]				X
Duke Social Support [67]				X
Edinburgh Postnatal Depression Scale [68]				X
Life Orientation Test [69]				X
Perinatal PTSD Questionnaire [70]				X

^1^Fixed choice and open-ended questions developed specifically for this study

### Outcome measures

#### Demographic data

To assess the comparability of the study groups, demographic and medical information will be collected from the parent and infant medical record.

#### Feasibility and acceptability of We3health™ and mFICare

To measure the feasibility and acceptability of We3health™, we will quantify the amount of parent usage of the application modules and their documentation of involvement with key care processes of infant caregiving, parenting skill acquisition and participation in the structured mFICare intervention (Phase 2 only). This will be compared with the level of involvement in these activities in the usual FCC condition. Specifically, we will use We3health™ to measure attrition from the study; hours spent at infant’s bedside; number of education sessions attended in-person, remotely or completed online; number of medical rounds sessions attended in-person or remotely; degree of active participation in medical rounds; amount of parent documentation completed; amount of participation in infant caregiving; skills acquired; and degree of independence in infant caregiving. To determine the acceptability of the mFICare intervention and of We3health™ data collection method, questionnaires will be administered to parents at discharge and to the clinical team as described above.

#### Primary outcome

To measure the primary outcome of infant growth, weight gain by 21 days of age will be obtained from the infant medical record and z-scores will be calculated. The z-score refers to the exact number of standard deviations greater or smaller than the median and is the standard recommended metric for infant growth studies [71]. Change in weight from entry into the study to discharge, and weight gain velocity by 21 days post-intervention will also be calculated to compare results with the FICare cRCT [46].

#### Secondary outcomes

Infant outcome measures are shown in Table 2. Secondary outcomes include: rate of human milk feeds at enrollment and discharge, time from birth to reaching full enteral feeds, length of NICU stay, and 5 major morbidities: (1) nosocomial infection [72]; (2) NEC [73]; (3) BPD [74]; (4) IVH [75]; and (5) ROP [76]. Number of adverse events for study infants and for the NICU per 1000 patient days for the usual FCC and mFICare enrollment periods will be compared. Parent stress will be assessed with multiple scales at different time points (see Table 1). Parents in the mFICare group will complete an additional FICare satisfaction questionnaire [25]. In the 3-month post-discharge survey we will also measure 1) infant growth, measured as change in z-scores and weight gain velocity from birth to 3 months after discharge; 2) proportion of infants receiving breastfeeding or human milk feeds; and 3) readmission rates.

**Table 2:** Infant measures

	Baseline	21 days after enrollment	Discharge	Post-discharge
Change in infant weight (z-score)		X		X*
Amount of human milk/formula supplementation	X		X	
Frequency of breastfeeding	X		X	X*
Breastfeeding rate	X		X	X*
Length of stay			X	
Weight gain velocity			X	
Major morbidities: Nosocomial infection, necrotizing enterocolitis, intraventricular hemorrhage, retinopathy of prematurity, and adverse events.			X	
Hospital readmission rate				X*

#### Clinical team outcomes

Overall NICU nurse and physician attitudes and perceptions about FCC will be measured using the validated Family Centered Care Questionnaire [77] at two time-points: Prior to completion of the usual FCC enrollment, and prior completion of the mFICare enrollment. Nurses and physicians caring for mFICare infants will also complete the FICare satisfaction questionnaire.

Intervention fidelity checks will be done by study personnel during the clinical team and alumni parent training and periodically throughout the mFICare study period. We will conduct regular site visits that will involve auditing of study recruitment procedures, intervention implementation and data collection. Site research staff will also participate in monthly calls to discuss study progress, address any questions about study operating procedures, address any concerns about protocol implementation, and share best practices.

### Statistical considerations

#### Sample size

We expect 225 eligible NICU admissions at the first study site during the study period and anticipate enrolling 50 parent-infant pairs in each group (usual FCC and mFICare) at that site. Assuming 20% attrition, we will have complete data on 40 infants per group. The study is exploratory at the other five sites to further assess feasibility and acceptability thus statistical power is based only on the first study site. The primary outcome, change in weight Z-score, typically has a standard deviation of between 0.44–0.47 (data from Toronto pilot and Canada-led cRCT). With a 0.05 null-hypothesis rejection threshold (alpha) for the two-tailed t-test, we will have 80% power to identify a group difference of 0.28–0.29.

### Data analysis

Descriptive statistics will be provided for all study variables: means and standard deviations for quantitative variables and frequencies and percentages for categorical variables. Data analysis will be based on an intention-to-treat strategy with all participants enrolled in the usual FCC or mFICare groups analyzed within their respective groups regardless of compliance with the group assignment. Outcomes will be compared between the usual FCC and mFICare groups using t-tests for continuous variables, Wilcoxon tests for ordinal variables, and chi-square tests for categorical variables. Differences among the groups in potential confounding variables (e.g., site, GA, birth weight) will be examined. Variables that differ among the groups may be considered as covariates when examining group differences in the primary and secondary outcomes [42, 46]. The covariate-adjusted scores may provide more precise estimates of the effect sizes. We will calculate confidence intervals around the differences because this will give a better idea of the range of values around the true means for the population. We will compare participation rates in specific aspects of usual FCC and mFICare such as caregiving activities, education, time spent with the infant or holding skin-to-skin or the primary and secondary outcome variables to see if they differ for parents and infants with different characteristics (e.g., sex, race/ethnicity or distance between hospital and home). We will compare attitudes and perceived practices of nurses and physicians during the FCC and mFICare conditions.

### Confidentiality and data security

Participants in the trial will be identified by a study ID only, with a master list linking names with numbers being held securely and separately from the study data. To ensure that all information is secure, data records will be kept in a secure location at each study site and on secure electronic systems (Salesforce™ (San Francisco, CA); Qualtrics™ (Provo, UT); REDCap™ (Fort Lauderdale, FL)) and accessible only to research staff. As soon as all follow-up is completed the data records will be de-identified. De-identified data will be used for the statistical analysis and all publications will include only aggregated data.

The electronic version of the data will be maintained on secure servers protected by password. All hard copy patient identifiable data and electronic backup files will be kept in locked cabinets, which are held in a locked room accessed only by key and limited staff. Data files will be stored for ten years after completion of the project as recommended by the University of California Records Retention Schedule. Disposal of identifiable information will be shredded.

## Discussion

High quality parent involvement in the care of NICU infants has longlasting positive effects on infant outcomes, particularly growth. However, the best methods for achieving this in the US context are not yet known. Results from this study will provide crucial data about the implementation of FICare in the US context within various hospital settings and identify important barriers, facilitators and key processes that may contribute to the effectiveness of FICare. It will also offer insights to clinicians on the feasibility of a new mobile application to support parent-focused research and greater accessibility to the FICare program to promote integration of parents into the NICU care team in US hospital settings.

Results from Phase 1 will provide useful data on current practices related to FCC, skin to skin, and breast feeding in the NICU setting. The Phase 1 results will also provide new information on the longitudinal experience of parents during and after their infant’s NICU admission and potentially uncover disparities in experience based on parent or infant characteristics. Evaluation of parent collaboration in co-designing the We3health™ mobile application and the study will help to inform future collaborative research between researchers and communities. Results from Phase 2 will additionally provide useful data on the differential uptake and effects of the FICare components adapted to the US setting and the uptake and response of parents who utilize the FICare resources remotely through the We3health™ mobile application. Finally, the tracking features of We3health™ will enable enhanced data capture of parent engagement and involvement in infant caregiving. We will be able to track measures such as parent time spent in the NICU and in infant care activities such as skin-to-skin care or breastfeeding. This will allow us to measure key processes of engagement and parent outcome measures for all parents entered into the study (FCC and mFICare groups). These measures are otherwise extremely burdensome to collect and lead to poor quality data. We will also be able to examine parent use of educational resources and peer support, preferences for information and engagement strategies, and we will be able to ensure data quality. The findings from this study will be communicated to participants upon request, and to healthcare professionals and NICU parent advocacy groups through presentations and publications.

## Data Availability

Not applicable.

## Abbreviations

FCC: Family Centered Care
FICare: Family Integrated Care
mFICare: Mobile enhanced Family Integrated Care
NICU: Neonatal Intensive Care Unit
